# Molecular Allergy Diagnostics in Children with Cow's Milk Allergy: Prediction of Oral Food Challenge Response in Clinical Practice

**DOI:** 10.1155/2023/1129449

**Published:** 2023-04-24

**Authors:** Maria Angela Tosca, Irene Schiavetti, Roberta Olcese, Chiara Trincianti, Giorgio Ciprandi

**Affiliations:** ^1^Allergy Center, IRCCS Giannina Gaslini, Genoa, Italy; ^2^Health Science Department, University of Genoa, Genoa, Italy; ^3^Allergy Clinic, Casa di Cura Villa Montallegro, Genoa, Italy

## Abstract

**Background:**

Cow's milk allergy (CMA) is the most common food allergy in early childhood. Children with CMA require a precise and punctual diagnosis. Oral food challenge (OFC) is the gold-standard procedure for diagnosing allergies, but it is laborious and requires a particular setting. The aim of the study was to identify the cutoff value of serum allergen-specific IgE values able to predict a positive response to OFC.

**Methods:**

Children with suspected CMA performed OFC with cow's milk (CM) or derivatives. Total IgE and specific IgE to raw CM, *α*-lactalbumin, *β*-lactoglobulin, and casein were measured.

**Results:**

Seventy-two children performed OFC, and 30 (41.6%) had a positive response. The significant predictive factors were sensitization to raw CM extract (*p* = 0.03), *α*-lactalbumin (*p* = 0.013), *β*-lactoglobulin (*p* = 0.09), and casein (*p* = 0.019). The cutoff was, respectively: 5.13 kUA/L for raw CM, 1.47 for *α*-lactalbumin, 1.35 for *β*-lactoglobulin, and 4.87 for casein.

**Conclusions:**

This study allowed us to define a set of cutoff values for CM protein-specific IgE. However, these cutoffs should be interpreted not as a diagnostic tool for CMA but only predictive of response to OFC in a specific territory. Thus, the practical message may be that a value above the cutoff allows a good approximation to identify children to be started on OFC.

## 1. Introduction

Food allergy represents a relevant health concern, mainly in childhood; in particular, cow's milk allergy (CMA) is the most relevant in infancy and early childhood, affecting up to 5% of children [1]. However, the actual prevalence significantly varies across countries as well as the severity of clinical manifestations [2]. Accordingly, a long-term UK study pointed to 23 children with CMA who had anaphylaxis after milk ingestion [3]. Therefore, CMA is a common, but not a trivial, medical condition.

From an immunological point of view, cow's milk (CM) consists of different allergenic molecules. The most relevant allergenic molecules are *α*-lactalbumin (Bos d 4), *β*-lactoglobulin (Bos d 5), and casein (Bos d 8). The sensitization prevalence of these molecules is hugely variable, whereas the allergy prevalence in sensitized subjects is about 20% [4]. In addition, it has to be underlined that boiling, pasteurization, ultra-high temperature treatment, evaporation, and formula maintain the milk allergenicity [5]. Only extensive hydrolysis significantly affects milk allergenicity. These clinically relevant concepts should be considered in CMA subjects with severe manifestations after milk or its derivatives ingestion.

Another essential characteristic of CMA is the natural history: the onset usually occurs before 12 months of age, at the same time as the introduction of CM [6]. Moreover, CMA commonly disappears over time as most children develop immunological tolerance to cow's milk. However, some subjects maintain CMA even during adulthood [7].

Symptoms usually occur immediately after CM ingestion and involve mainly the skin, gastrointestinal and respiratory tract, and, more rarely, the cardiovascular system [8]. Furthermore, symptoms intensity may range from mild to severe until anaphylactic shock, also fatal [9].

Consequently, CMA patients require adequate management based on thorough workup and close follow-up. Namely, an early and precise diagnosis is mandatory for prescribing a tailored diet and avoiding useless and potentially dangerous milk avoidance. The correct diagnosis of CMA requires a suggestive history, the documentation of sensitization, i.e., production of specific IgE, and consistency between history and sensitization [10].

Sensitization may be investigated by skin prick test, with allergen extracts or fresh milk, and/or serum IgE assay. However, serum assay is more precise and reliable than skin testing [11]. Moreover, serum testing presently is advantaged by the use of molecular diagnostics, which allows the identification of the immunological profile in each patient [12]. In this regard, the most recent guidelines on managing CMA patients reported the diagnostic criteria for interpreting the laboratory outcomes [13–18]. However, it must be emphasized that only the oral food challenge (OFC) makes it possible to acquire a definite diagnosis of CMA [13–18].

Though, OFC should be performed only in particular settings, characterized by trained staff and equipped with adequate tools. Therefore, predictive tools that facilitate CMA suspicion can be handy in clinical practice. In this regard, several studies have attempted to define cutoffs of specific IgE levels, mainly molecular-specific, that could identify subjects with suspected CMA as a first step [19–28]. However, the reported values needed to be more consistent. Based on this background, the present study aimed to evaluate the cutoff of allergen-specific IgE to milk and its molecules in clinical practice.

## 2. Materials and Methods

This study retrospectively evaluated all children consecutively admitted to performing OFC from 2013 to 2022. The data were extracted from an electronic platform containing demographics, clinical history, and biological data.

Laboratory data included peripheral eosinophils, total serum IgE, and allergen-specific IgE to casein, *α*-lactalbumin, *β*-lactoglobulin, and raw milk.

IgE was measured using ELISA assays provided by Thermofisher (Milan, Italy). The total serum IgE normal level is <100 kU/L. Sensitization is defined when the value of allergen-specific IgE is >0.35 kUA/L.

The inclusion criteria were the need to perform OFC for suspected CMA, suggested by symptom occurrence after CM ingestion, sensitization to CM extract and/or milk molecules, and pediatric age. Exclusion criteria were concomitant diseases and medications that could interfere with interpreting results.

The OFC was performed using the following procedure: patients started from doses between 0.05 and 1 mL, progressively incremented. The OFC ended when a clinical manifestation occurred or a cumulative dose of 80 mL had been reached.

The Ethics Committee of the IRCCS Istituto Giannina Gaslini approved the procedure (10/17; 04/05/2017). Parents signed informed consent.

A univariate logistic regression model was applied to identify which variables best predict response after OFC. Subsequently, a receiver operating characteristic curve was used to assess the accuracy of different IgE classes in predicting the response. For each receiver operating characteristic curve, the area under the curve with the corresponding 95% confidence interval was calculated with the best cutoff point, which provided both the highest sensitivity and specificity.

A 2-tailed *p* value of 0.05 was considered significant. All statistical analyses were performed using SPSS 23.0 (SPSS, Chicago, Illinois).

## 3. Results

Seventy-two children, 52 males (72.2%) and 20 females (27.8%), mean age of 71.6 months, performed the OFC to CM, as reported in Table 1. Fifty (69.4%) children were sensitized to other food allergens and 43 (59.7%) to inhalant allergens. In addition, different comorbidities were present: 30 (41.7%) children had atopic dermatitis, 38 (52.8%) recurrent wheezing, 21 (29.2%) allergic rhinitis, 5 (6.9%) chronic urticaria, 1 (1.4%) oral allergy syndrome. Forty (55.6%) children had at least one family member with an allergy.

The mean total IgE level was 476.4 kU/L, the data concerning sensitization to milk and its molecules are in detail reported in Table 1.

Thirty (41.6%) children were positive for OFC with milk (Table 2). The analysis concerned the valuable parameters that could predict the positive response to OFC. The significant predictive factors were sensitization to raw CM extract (*p* = 0.03), *α*-lactalbumin (*p* = 0.013), *β*-lactoglobulin (*p* = 0.09), and casein (*p* = 0.019). The cutoff was, respectively: 5.13 kUA/L for raw CM, 1.47 for *α*-lactalbumin, 1.35 for *β*-lactoglobulin, and 4.87 for casein. In addition, area under the curve (AUC), specificity, and sensitivity values are reported in detail in Table 2.

## 4. Discussion

CMA is the most frequent food allergy in children under the age of 3 years. Children with CMA require hardworking management by the physician and constant attention from the parents. However, it is quite common to observe children on unnecessary and often harmful exclusion diets at an age when milk is an essential nutrient for growth. Therefore, a precise and accurate diagnosis is the fundamental premise for properly managing CMA. In this regard, OFC remains the gold standard for providing a certain CMA diagnosis [20]. However, OFC is a laborious and potentially risky procedure that should be performed only in highly qualified centers. As a result, children undergoing OFC need a rigorous selection based on a reasonable presumption of CMA. A suggestive history and sensitization to milk molecules may predict CMA with good approximation [10]. Therefore, data on sensitization to CM allergens could represent a valuable tool to corroborate a suspicion of CMA and thus refer a child to the OFC. In this regard, several studies attempted to define a reliable cutoff of specific IgE levels to predict a positive response to OFC and, consequently, identify children with CMA [29].

The present study derived from previous research showing that children with anaphylaxis to CM proteins had higher levels of specific IgE than children with mild CMA [10]. In particular, children with >12.2 kUA/L IgE to casein had an OR of 15 to have anaphylaxis compared to children with IgE levels below this cutoff.

As we recently reported data concerning children with CMA and undergoing oral immunotherapy [29], the present study aimed to identify factors predictive for positive OFC and define the cutoff of specific IgE levels.

The results highlighted that the predictive cutoff for raw CM was 5.13 kUA/L, 1.47 for *α*-lactoglobulin, 1.35 for *β*-lactalbumin, and 4.87 for casein. However, these cutoff values are only partially reliable considering the single AUC scores, specificity, and sensitivity outcomes. Moreover, these findings are partially consistent with the most recent literature data, as reported in Table 3. Namely, a Brazilian study reported a cutoff of 3.06 kUA/L for CM (sensitivity 71%; specificity 98%), 2.08 for *α*-lactalbumin (sensitivity 58%; specificity 98%), 1.85 for *β*-lactoglobulin (sensitivity 57%; specificity 98%), and 1.47 (sensitivity 66%; specificity 98%) [22]. A Danish study identified different cutoff values for milk proteins: 3.64 for raw CM, 0.77 for *α*-lactalbumin, 1.59 for *β*-lactoglobulin, and 2.33 for casein [25]. A Spanish study reported the following cutoff values: 3.87 for raw CM, 2.25 for *α*-lactalbumin, 1.6 for *β*-lactoglobulin, and 0.95 for casein [27]. Finally, a Portuguese study identified these values: 7 for raw CM, 8.8 for *α*-lactalbumin, 7 for *β*-lactoglobulin, and 7.3 for casein [28]. In addition, Cuomo et al. [24] reviewed 31 studies on this topic and identified the cutoff of 5 kUA/L for CM extract as a likely predictor for CMA.

Therefore, there is no complete agreement between the various cutoffs defined by the various studies. In addition, considering the criteria for validity (e.g., AUC, sensitivity, specificity), all the more reason that each study population has different outcomes. Consequently, the proposed cutoff values can have a somewhat relative reference value primarily because of the country under consideration. In this regard, the relative inconsistency among these studies could depend on different populations and the lack of sIgE/tIgE relationships for a more precise analysis.

Therefore, the results should always be interpreted cautiously and related to the survey location. Furthermore, based on the validity criteria of the tests, it seems necessary to emphasize that the diagnosis of CMA mandatorily requires the performance of OFC.

This study had several limitations, especially the limited number of subjects and the failure to perform a double-blind OFC with a placebo. In addition, the ratio between total IgE and allergen-specific IgE was not performed. On the other hand, this study reflects what can occur in real-life in a level 3 pediatric allergy center.

In conclusion, this study allowed us to define a set of cutoff values for CM protein-specific IgE. However, these cutoffs should be interpreted not as a diagnostic tool for CMA but only predictive of response to OFC in a specific territory. Thus, the practical message may be that a value above the cutoff allows a good approximation to identify children to be started on OFC.

## Figures and Tables

**Table 1:** Baseline characteristics (*n* = 72).

Sex	
Females	20 (27.8%)
Males	52 (72.2%)
Age (months)	71.6 ± 56.61
Sensitisation to other food allergens	50 (69.4%)
Sensitisation to inhalant allergens	43 (59.7%)
Atopic dermatitis	30 (41.7%)
Recurrent wheezing	38 (52.8%)
Allergic rhinitis	21 (29.2%)
Chronic urticaria	5 (6.9%)
Oral allergy syndrome	1 (1.4%)
Other food allergies	17 (23.6%)
Allergy in family	40 (55.6%)
Allergy tests	
Total IgE (kU/L)	476.4 ± 733.0
IgE to cow's milk (kUA/L)	23.2 ± 35.84
IgE to Bos d 4 (*α*-lactalbumin) (kUA/L)	13.4 ± 26.96
IgE to Bos d 5 (*β*-lactoglobulin) (kUA/L)	12.8 ± 23.35
IgE to Bos d 8 (casein) (kUA/L)	16.4 ± 27.50

**Table 2:** Predictive factors for a positive response to CM challenge.

	Negative challenge (*n* = 42; 58.4%)	Positive challenge (*n* = 30; 41.6%)	*p*	AUC (95% CI)	Cutoff	Spec.	Sens.
Age (months)	60.8 ± 49.81	86.7 ± 62.71	0.05				
Sex							
Females	11 (26.2%)	9 (30.0%)	0.79				
Males	31 (73.8%)	21 (70.0%)					
Severity of reaction							
Mild	9 (23.1%)	10 (41.7%)	0.27				
Moderate	15 (38.5%)	6 (25.0%)					
Severe	15 (38.5%)	8 (33.3%)					
Total IgE (kU/L)	605.7 ± 1227.47	661.4 ± 1041.39	0.65				
IgE to cow's milk (kUA/L)	8.4 ± 14.72	22.8 ± 30.89	0.03	0.65 (0.52–0.79)	5.13	70.0%	65.5%
IgE to Bos d 4 (*α*-lactalbumin) (kUA/L)	2.8 ± 5.76	11.1 ± 21.66	0.013	0.68 (0.55– 0.81)	1.47	71.1%	62.1%
IgE to Bos d 5 (*β*-lactoglobulin) (kUA/L)	2.8 ± 5.62	6.5 ± 12.68	0.09	0.62 (0.49–0.76)	1.35	67.6%	58.6%
IgE to Bos d 8 (casein) (kUA/L)	5.5 ± 10.74	18.5 ± 27.57	0.019	0.67 (0.54–0.81)	4.87	81.6%	51.9%
Total daily cumulative dose administered on the last day of the challenge (mL)	23.7 ± 33.03	27.2 ± 43.79	0.17				

**Table 3:** Predictive cutoff values for raw caw's milk (*F* 2), *α*-lactoalbumin (Bos d 4), *β*-lactoglobulin (Bos d 5), and casein (Bos d 8) reported in the most recent literature. Data are expressed as kUA/L.

Author	Reference	*F* 2	Bos d 4	Bos d 8	Bos d 5
Castro et al.	[22]	3.06	2.08	1.85	1.47
Cuomo et al.	[24]	≥5			
Petersen et al.	[25]	3.64	0.77	1.59	2.33
Ayats-Vidal et al.	[27]	3.87	2.25	1.6	0.95
Castro Neves et al.	[28]	7	8.8	7	7.3
Present study		5.13	1.47	1.35	4.87

## Data Availability

Data are available on demand to the corresponding author.
